# Turkish Adaptation and Psychometric Evaluation of the Hospital Consumer Assessment of Healthcare Providers and Systems (HCAHPS) Survey: A Multicenter Study

**DOI:** 10.1111/nhs.70391

**Published:** 2026-07-15

**Authors:** Mehmet Gülşen, Hasan Fehmi Dirik, Kubilay Kaymaz, Asuman Kuşçu, Melis Yazici, Işıl Yerlikaya, İlkay Baylam

**Affiliations:** ^1^ İvrindi Vocational School of Health Services, Balıkesir University Balıkesir Turkey; ^2^ Faculty of Nursing Dokuz Eylül University İzmir Turkey; ^3^ American Hospital İstanbul Turkey; ^4^ Anadolu Medical Center Kocaeli Turkey; ^5^ Ankara Güven Hospital Ankara Turkey; ^6^ Planetree International Derby Connecticut USA

**Keywords:** patient experience, questionnaire, reliability, survey, validity

## Abstract

Patient experience is a critical indicator of healthcare quality and therefore requires assessment using robust measurement tools. One such is the Hospital Consumer Assessment of Healthcare Providers and Systems (HCAHPS) survey. However, limited evidence supports its validation, underscoring the need for further psychometric evaluation across diverse healthcare contexts. A multicenter cross‐sectional psychometric study was conducted across three private hospitals in Turkey. Between December 2023 and December 2024, data were collected from 478 patients following hospital discharge. Content validity was assessed using content validity indices, and construct validity was examined through confirmatory factor analysis. Reliability was assessed using Cronbach's alpha and McDonald's omega. The Turkish HCAHPS maintained its original structure with excellent content validity. Confirmatory factor analysis demonstrated a good model fit (*χ* ^2^/df = 2.06; RMSEA = 0.05; CFI = 0.98; NFI = 0.96). Reliability analysis revealed that both Cronbach's alpha and McDonald's omega values ranged from 0.71 to 0.95, indicating acceptable to excellent internal consistency across composites. The Turkish version of the HCAHPS demonstrated acceptable psychometric properties, supporting its use as a reliable tool for measuring patient experience, with potential to inform quality improvement efforts in hospital settings.

## Summary

- Patient experience is a critical indicator of healthcare quality and can be assessed through standardized instruments, such as the HCAHPS survey; however, cultural and linguistic adaptations are essential for accurate measurement in different contexts.
- This study provides the first rigorous translation, cultural adaptation, and psychometric validation of the HCAHPS survey for the Turkish healthcare setting.
- The validated Turkish version demonstrates strong reliability and validity, enabling accurate assessment of patient experiences in Turkish hospitals.

## 1 Introduction

Patient experience is a critical indicator of healthcare quality, offering valuable insights into the patient's perspective of the effectiveness of care delivery. The Beryl Institute (2025) defines patient experience as “the sum of all interactions, shaped by an organization's culture, that influence patient perceptions across the continuum of care.” Understanding and improving patients' experiences is important in enhancing the quality of care, fostering trust, and facilitating their communication with healthcare providers. Similarly, according to the Agency for Healthcare Research and Quality [AHRQ] (2025), patient experience encompasses the range of patient interactions with the healthcare system. This includes not only the care patients receive from health plans, doctors, nurses, and other staff in hospitals but also from physician practices and other healthcare facilities.

In recent years, healthcare providers and policymakers have shown growing interest in measuring patients' views of their hospital care. Healthcare institutions' primary goal is providing optimum care, which can be best achieved by regarding patients as partners who are actively involved in the processes and evaluation of care (Wolf et al. 2021). This growing emphasis on assessment has increased demand for tools that can credibly assess inpatient experience and support quality improvement initiatives. Unfortunately, however, Turkey lacks standardized inpatient experience measures with published evidence on translation, cultural adaptation, and psychometric performance.

As patient experience is not directly observable, it is often measured using surveys, transforming subjective feedback into meaningful, quantifiable, and actionable data (NEJM Catalyst 2018). There are three widely used tools to assess patient experiences. The Hospital Consumer Assessment of Healthcare Providers and Systems (HCAHPS) survey is one of the most widely used standardized instruments for assessing inpatient experience (Bernardo et al. 2022). Developed in the United States by the Centers for Medicare and Medicaid Services (CMS) in partnership with the Agency for Healthcare Research and Quality (AHRQ 2025), the HCAHPS survey evaluates various dimensions of patient experience, including communication with healthcare providers (physicians and nurses), staff responsiveness, information on medicines, discharge procedures, and the overall hospital environment (HCAHPS 2025).

HCAHPS has been translated, culturally adapted, and psychometrically validated in several non‐English contexts, including Japan (Aoki et al. 2020), Malaysia (Zun et al. 2019), the Philippines (Judan‐Ruiz et al. 2020), and Saudi Arabia (Alanazi et al. 2017). This growing body of evidence indicates that the instrument can be meaningfully adapted across diverse linguistic and cultural settings. HCAHPS' continued relevance is further reflected in its use as a measurement tool in recent evidence reviews that synthesize interventions, care processes, and hospital characteristics associated with improved inpatient experience (Beckett et al. 2024; Hurwitz et al. 2023). Another of its uses is the evaluation of communication‐focused quality improvement initiatives (Tiperneni et al. 2022), assessing inpatient experience during system‐level disruptions such as the COVID‐19 pandemic (Elliott et al. 2023), and examining factors associated with patient satisfaction at the national level (Liang et al. 2021).

The second most frequently used instrument in this area is the Picker Patient Experience Questionnaire (Bernardo et al. 2022). Created by Reeves et al. (2002), it focuses on information availability, care coordination, physical comfort, emotional support, respect for patient preferences, family involvement, continuity of care, and overall patient perception. The third commonly utilized instrument is the Quality of Care from the Patient's Perspective (Bernardo et al. 2022), developed by Wilde et al. (1993), focused on assessing factors of physician competence, technical conditions, and the sociocultural environment within healthcare settings. A systematic review of patient experience tools emphasizes that there is no ‘one‐size‐fits‐all’ approach to selecting an instrument for measuring the patient experience of hospital quality of care. However, for high‐stakes purposes, such as improvement incentives or research, choosing a highly reliable tool, such as the HCAHPS survey, is essential (Beattie et al. 2015). In addition to this recommendation, a scoping review identified HCAHPS as potentially the most appropriate instrument (Bernardo et al. 2022); therefore, it was selected for its recency and the comprehensive range of issues covered, including the key areas of nursing care, medication safety, and discharge procedures. Furthermore, in Turkey, as in many other countries, there is an increasing emphasis on patient‐centered care and the need for reliable tools to evaluate healthcare services. The translation and adaptation of this internationally validated and widely used tool is expected to contribute significantly to evaluating inpatient care.

### 1.1 Aim

The present study aimed to translate, culturally adapt, and validate the HCAHPS survey for use in Turkish hospitals and contribute to the internationality of the validation and reliability of the HCAHPS.

## 2 Method

### 2.1 Study Design

This psychometric study employed a cross‐sectional design and was conducted in two phases: (1) the translation and language adaptation of the survey and (2) the administration of the survey. Translating and adapting instruments for the specific context require careful planning and taking a sound methodological approach. To ensure compliance with these standards, we reported the scale's psychometric and psycholinguistic properties according to three sets of guidelines: COSMIN (Mokkink et al. 2010, 2012), ISPOR (Wild et al. 2005), and STROBE (von Elm et al. 2007).

### 2.2 Setting

The study was conducted in three private hospitals in different cities across Turkey. The first, in Istanbul, is a tertiary care facility with 278 patient rooms. It serves over 220 000 patients annually, performing more than 10 000 surgeries. The second hospital, in Kocaeli, is a multidisciplinary center offering services in various branches of medicine. It has a capacity of 201 beds and is recognized for its strategic partnerships and international accreditations. The third hospital, in Ankara, is a general hospital with 254 beds. Hospitals from different regions were selected to provide a range of data and support the generalizability of the findings (Mokkink et al. 2012).

### 2.3 Sampling and Participants

Patients included in the study were 18 years or older with at least one overnight hospital stay. The following groups were excluded: pediatric patients (under 18 years old at the time of admission) and those with a primary psychiatric or substance abuse diagnosis. This was because the current HCAHPS Survey is designed to address neither the unique situation of pediatric patients and their families nor the behavioral health issues pertinent to psychiatric patients.

Guidance summarized by Carpenter (2018) indicates that many authors recommend a minimum sample size of 300 for factor‐analytic studies and minimum participant‐to‐item ratios of at least 5:1 (often 5:1–10:1), with higher ratios suggested for more robust solutions. The final sample of 478 participants exceeded these commonly cited thresholds, supporting the adequacy of the sample size for the planned psychometric analyses.

### 2.4 Measurement

The HCAHPS Survey is a standardized tool designed to evaluate hospital patient experiences, consisting of 29 items (HCAHPS 2025). The first 22 items are as follows: Communication with nurses (Questions 1–3), communication with doctors (Questions 5–7), responsiveness of hospital staff (Questions 4 and 11), hospital environment (Questions 8 and 9), communication about medicines (Questions 13 and 14), discharge information (Questions 16 and 17), hospital rating (Question 18), willingness to recommend the hospital (Question 19), and care transition (Questions 20–22). Additionally, the survey contains items such as whether they were admitted through the Emergency Room (Question 23), their general health, mental and emotional health (Questions 24 and 25), and educational level (Question 26). The original survey also includes demographic questions (items 27–29) regarding race, ethnic identity, and the language spoken at home, but these were considered contextually and conceptually irrelevant for the current context and were therefore excluded from the Turkish version.

Two of the experience‐related questions are dichotomous (yes/no) items that evaluate discharge preparedness, expressly, whether relevant post‐discharge information was provided. The remaining items are scored on a four‐point scale ranging from “never” to “always.” The two global care ratings are based on a 0 to 10 scale for the overall hospital rating (where 0 represents the worst hospital possible) and a four‐point scale for the willingness to recommend the hospital (where one equals “definitely no” and four equals “definitely yes”).

In the initial instrument development study, the items were refined through cognitive testing to improve comprehensibility and strengthen content validity (Levine et al. 2005). Subsequently, psychometric analyses used in a multi‐state pilot study supported the instrument's multidomain structure and also provided evidence of reliability, construct validity, and criterion‐related validity for hospital‐level comparisons. Internal consistency coefficients for the shortened composite measures ranged from 0.51 to 0.88 across domains (Keller et al. 2005). Nevertheless, with the expansion of the implementation of HCAHPS, more recent research has highlighted the need for additional psychometric evaluation across diverse settings and populations (Westbrook et al. 2014).

### 2.5 Data Collection

Online surveys were used to collect data across three private hospitals located in different Turkish cities between December 2023 and December 2024. A total of 492 submissions were received; 14 submissions containing only sociodemographic information without HCAHPS item responses were excluded prior to analysis, yielding a final analytic sample of 478 patients. The participating hospitals were in İstanbul, Ankara, and Kocaeli, which are among Turkey's most populous provinces. The hospitals represented were all large‐scale private providers with inpatient capacity exceeding 200, and all held Joint Commission International accreditation and Planetree person‐centered care certification. The survey administration was conducted within a period extending from 48 h to 6 weeks (42 calendar days) following discharge.

Authorized hospital staff used hospital discharge records to identify eligible patients. The survey link was subsequently distributed via short message service SMS or email using the contact information provided at discharge. The message included a brief study invitation, an information sheet, and an electronic consent statement; participants accessed the questionnaire on their own devices by clicking the link and completing it via self‐administration. Before analysis, submissions were screened for potential duplicate responses based on similarities in completion times and/or response patterns.

### 2.6 Statistical Analysis

Data analysis was conducted using IBM SPSS (version 26, Chicago, IL, USA) and AMOS (version 21, Chicago, IL, USA). Fourteen questionnaires (2.85%) with no HCAHPS item responses were excluded using listwise deletion; the resulting risk of sample bias was considered minimal. Descriptive statistics such as means and standard deviations were used for measurement data, while frequencies and percentages were employed for count data. The validity of the Turkish HCAHPS was assessed through content, construct, and criterion‐related validity. Expert panel evaluations guided content validity, and scores were used for content validity indices. Construct validity was assessed using confirmatory factor analysis (CFA). The four‐point Likert‐type items were retained in their original ordered response metric and entered the model as observed item scores, whereas the yes/no discharge information items were entered as dichotomous observed indicators. The model parameters were estimated using maximum likelihood estimation based on the observed covariance matrix. The following fit indices were utilized to evaluate the model fit: *χ* ^2^/df (≤ 2 good, ≤ 3 acceptable), RMSEA (≤ 0.05 good, ≤ 0.08 acceptable), NFI (≥ 0.95 good, ≥ 0.90 acceptable), CFI (≥ 0.97 good, ≥ 0.95 acceptable), GFI (≥ 0.95 good, ≥ 0.90 acceptable), AGFI (≥ 0.95 good, ≥ 0.85 acceptable) (Schermelleh‐Engel et al. 2003). HCAHPS composite scores and the overall hospital rating item (Q18) were used to examine criterion‐related validity, consistent with prior HCAHPS adaptation studies (Aoki et al. 2020). The scale's reliability was assessed through internal consistency using Cronbach's alpha and McDonald's omega, supported by corrected item‐total score correlations. Cronbach's alpha values of > 0.70 indicated a satisfactory result (Nunnally and Bernstein 1994). A threshold of 0.30 was established for item‐total score correlations (Boateng et al. 2018). Furthermore, item discrimination for each item was evaluated using extreme‐groups comparisons between the upper and lower 27% of respondents.

### 2.7 Translation and Cultural Adaptation

The translation of the HCAHPS survey was conducted following guidelines in the CAHPS Translating Surveys document provided by the AHRQ (2016). The process involved taking several key measures to ensure cultural and linguistic accuracy. First, we obtained two independent forward translations of the original English survey, produced by professional translators who were native speakers of the target language and experienced in health‐related translations. These translations were then assessed by the authors for accuracy, linguistic quality, and cultural appropriateness. This process was facilitated by the creation of a master table containing columns for the original English text, the two forward translations, and a reconciled version, and also a section for the reviewer's comments and notes on suggested revisions. The reconciliation process was carried out exclusively by three members of the research team, who, after reviewing the original English text, the two forward translations, and a reconciled version, reached a consensus on the final survey translation. Throughout the process, the following considerations were prioritized: ensuring readability by using plain and standard language, considering regional language variations, and adjusting for the target population's socio‐demographic characteristics. This rigorous approach, including the systematic use of the master table, ensured that the translated survey maintained both conceptual and cultural equivalence with the original instrument. To improve clarity and cultural appropriateness, selected items underwent minor semantic refinement; for example, the term “call button” was replaced with “call bell,” more common in the local context. Additionally, the wording of frequency items was standardized to ensure linguistic consistency. Back‐translation was then performed by two independent translators unfamiliar with the original items. A pilot test involved 30 patients, 10 recruited from each hospital, who provided feedback on the item's comprehensibility and clarity. They reported no problematic items or negative feedback, so no further revisions were necessary.

### 2.8 Ethical Considerations

Permission to translate the HCAHPS into Turkish was first obtained from the AHRQ CAHPS Program and the U.S. Centers for Medicare & Medicaid Services (CMS). Subsequently, ethical approval for this study was granted by the non‐interventional research ethics committee of Balıkesir University under approval number 2023/29. Written permissions to administer the survey were also obtained from the hospitals. Prior to discharge, eligible patients were informed that they might be invited to complete a patient experience survey after discharge, and their permission to be contacted was obtained. The questionnaire was self‐administered electronically, and responses were collected without direct personal identifiers. Patients were informed that participation was voluntary and that non‐participation would not affect their care or their relationship with the hospital. All participants provided electronic informed consent before completing the questionnaire.

## 3 Results

### 3.1 Characteristics of the Participants

Table 1 shows the participants' characteristics. The mean age was 47.64 years (±18.18). The majority (54.4%) were under age 50, while 45.6% were 50 or older. Gender distribution showed that 58.2% of the participants were female and 41.8% were male. Educational attainment varied, with 43.7% holding a bachelor's degree and 25.1% having a postgraduate degree. Smaller percentages had completed education to primary school (3.6%), middle school (2.1%), high school (17.8%), or earned an associate degree (7.7%). Regarding hospitalization context, 14.2% of participants reported admission via the emergency department (*n* = 68).

**TABLE 1:** Characteristics of the participants (*n* = 478).

Variables	*n*	%
Age	47.64 (18.18)	
< 50 years	260	54.4
≥ 50 years	218	45.6
Gender		
Male	200	41.8
Female	278	58.2
Education level		
Primary school	17	3.6
Middle school	10	2.1
High school	85	17.8
Associate degree	37	7.7
Bachelor's degree	209	43.7
Postgraduate degree	120	25.1
Chronic disease state		
Yes	141	29.5
No	337	70.5
Length of stay		
< 2 days	244	51.0
≥ 2 days	234	49.0
Admission via emergency department		
Yes	68	14.2
No	410	85.8
Overall health rating		
Excellent	35	7.3
Very good	144	30.1
Good	239	50.0
Fair	57	11.9
Poor	3	0.6
Mental or emotional health rating		
Excellent	65	13.6
Very good	174	36.4
Good	197	41.2
Fair	35	7.3
Poor	7	1.5

Regarding disease status, 29.5% of participants reported a chronic disease. The length of stay in the healthcare facility was almost equally divided, with 51.0% staying for less than 2 days, and 49.0% staying for 2 days or more.

In terms of overall health ratings, 50.0% of participants rated their health as good, 30.1% as very good, and 7.3% as excellent. 11.9% rated their health as fair, while a small group rated it as poor (0.6%). Mental or emotional health ratings showed that 41.2% rated their mental health as good, 36.4% as very good, and 13.6% as excellent, with fewer rating it as fair (7.3%) or poor (1.5%).

### 3.2 Validity

A panel of nine experts was selected for the content validity assessment in this study, consisting of six nursing academicians with experience in scale adaptation and proficiency in English, one midwifery academician, one clinical nurse, and one patient rights unit manager. These experts' scores were used to calculate the item content validity index (I‐CVI) and scale content validity index (S‐CVI). The I‐CVI was determined by identifying the number of experts who scored 3 (quite relevant) or 4 (highly relevant) and dividing this number by the total number of participating experts. Similarly, the S‐CVI/Ave was calculated as the mean of I‐CVI for all scale entries. Generally, I‐CVI ≥ 0.78 and S‐CVI/Ave ≥ 0.90 were considered acceptable (Polit and Beck 2006). In the present study, the I‐CVI values ranged from 0.89 to 1.00 across the Turkish HCAHPS items, and the S‐CVI/Ave was 0.97. These findings indicated that all I‐CVI and S‐CVI/Ave values exceeded the recommended thresholds. Accordingly, the Turkish HCAHPS retained its original structure following the content validity assessment.

**FIGURE 1:**
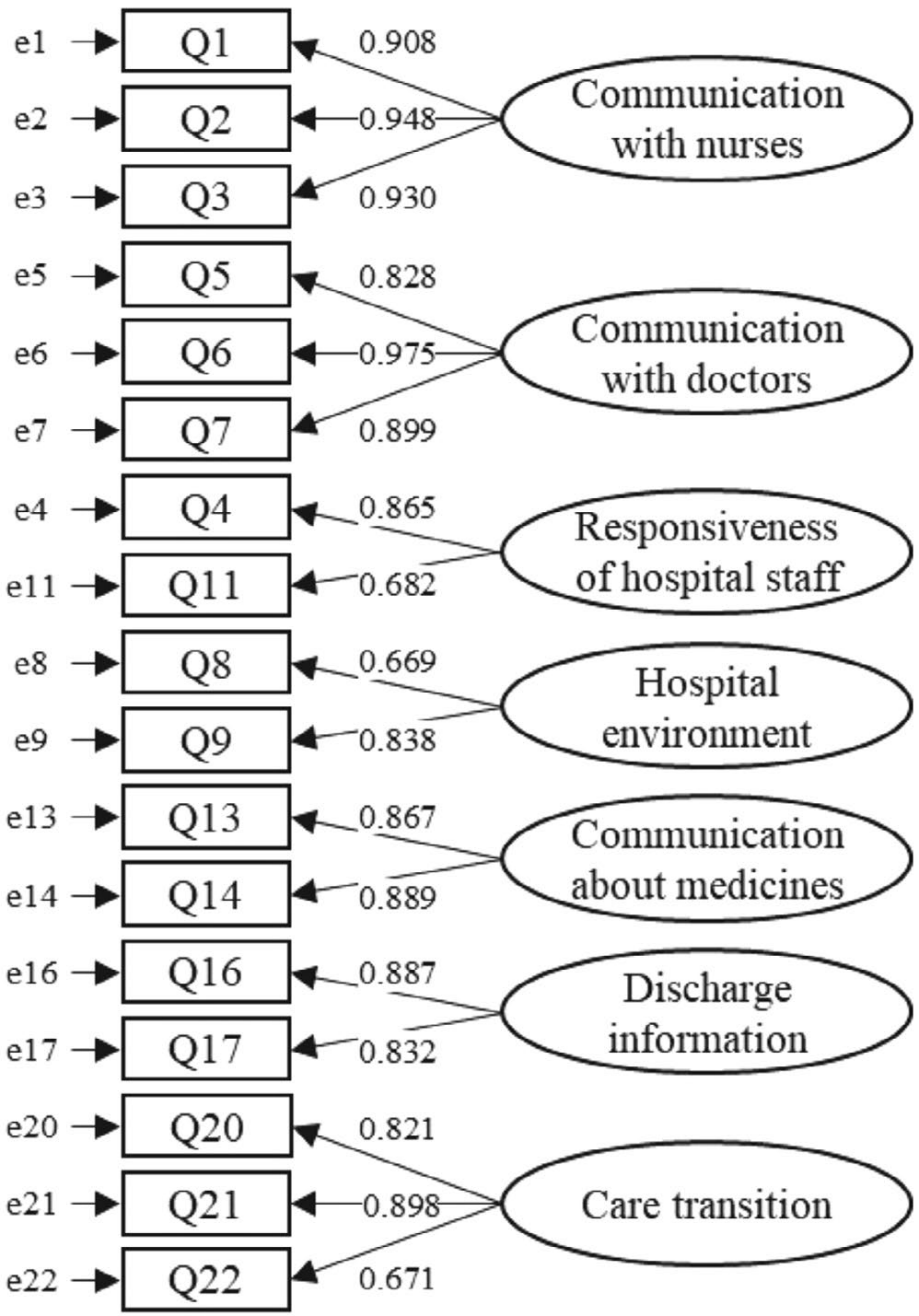
Factor structure of Turkish HCAHPS with standardized factor loadings.

CFA was employed to assess the structure of the Turkish HCAHPS. In this study, the model showed good fit with *χ* ^2^/df = 2.06, RMSEA = 0.05, NFI = 0.96, CFI = 0.98, GFI = 0.95, AGFI = 0.92 (Table 2). Item‐level CFA results are reported in Table 3. Standardized factor loadings were moderate to very strong, ranging from 0.669 to 0.975, and all freely estimated loadings were statistically significant (*p* < 0.001). Squared multiple correlations indicated that the latent factors explained 44.7% to 95.1% of the variance in item responses (SMC = 0.447–0.951). The final model with standardized loadings is presented in Figure 1.

**TABLE 2:** Confirmatory factor analysis fit indices.

Fit indices	Good fit	Acceptable fit	Present study
*χ* ^2^/df	≤ 2	≤ 3	2.06
RMSEA	≤ 0.05	≤ 0.08	0.05
NFI	≥ 0.95	≥ 0.90	0.96
CFI	≥ 0.97	≥ 0.95	0.98
GFI	≥ 0.95	≥ 0.90	0.95
AGFI	≥ 0.95	≥ 0.85	0.92

*Note:* Cut‐offs were based on Schermelleh‐Engel et al. (2003).

Abbreviations: *χ* ^2^/df = Chi‐square minimum/degree of freedom; AGFI = Adjusted Goodness‐of‐Fit‐Index; CFI = Comparative Fit Index; GFI = Goodness of Fit Index; NFI = Normed Fit Index; RMSEA = Root Mean Square Error of Approximation.

**TABLE 3:** Item‐level CFA results.

Composite	Item	*B*	SE	CR	*p*	*β*	SMC (*R* ^2^)
Communication with nurses	Q1	0.857	0.025	34.359	< 0.001	0.908	0.824
	Q2	0.967	0.025	39.055	< 0.001	0.948	0.898
	Q3	1.000	—	—	Fixed	0.930	0.866
Communication with doctors	Q5	0.719	0.028	25.586	< 0.001	0.828	0.686
	Q6	1.070	0.031	34.904	< 0.001	0.975	0.951
	Q7	1.000	—	—	Fixed	0.899	0.808
Responsiveness of hospital staff	Q4	1.371	0.094	14.603	< 0.001	0.865	0.749
	Q11	1.000	—	—	Fixed	0.682	0.465
Hospital environment	Q8	0.709	0.055	12.898	< 0.001	0.669	0.447
	Q9	1.000	—	—	Fixed	0.838	0.703
Communication about medicines	Q13	0.685	0.047	14.668	< 0.001	0.867	0.751
	Q14	1.000	—	—	Fixed	0.889	0.790
Discharge information	Q16	1.125	0.075	14.911	< 0.001	0.887	0.786
	Q17	1.000	—	—	Fixed	0.832	0.692
Care transition	Q20	1.353	0.088	15.418	< 0.001	0.821	0.674
	Q21	1.364	0.084	16.161	< 0.001	0.898	0.806
	Q22	1.000	—	—	Fixed	0.671	0.450

*Note:* One indicator per latent factor was fixed to 1.00 for identification; therefore SE, CR, and p are not estimated for fixed loadings. All freely estimated loadings were statistically significant (*p* < 0.001).

Abbreviations: *β* = standardized factor loading; *B* = unstandardized factor loading; CR = critical ratio; SE = standard error; SMC (*R* ^2^) = squared multiple correlation.

Criterion‐related validity evidence was examined by correlating Turkish HCAHPS composite scores with the overall hospital rating item and was found to be significantly positive (*r* = 0.813, *p* < 0.01). In hospital‐stratified analyses, the association between HCAHPS scores and the overall hospital rating remained consistently positive across hospitals (*r* = 0.784–0.937), supporting criterion‐related validity within each care setting.

### 3.3 Reliability

Table 4 shows descriptive features and internal consistency reliability of Turkish HCAHPS. The mean score of composite measures communication with nurses was 3.70 ± 0.61, communication with doctors was 3.82 ± 0.48, responsiveness of hospital staff was 3.42 ± 0.60, hospital environment was 3.67 ± 0.59, communication about medicines was 3.69 ± 0.69, and discharge information was 1.77 ± 0.38. The overall rating of the hospital had a mean of 8.53 ± 2.35. The willingness to recommend the hospital had a mean of 3.60 ± 0.79. Internal consistency was acceptable; both Cronbach's alpha and McDonald's omega coefficients ranged from 0.71 to 0.95 across composites. Corrected item‐total correlation values of the scale ranged between 0.484 and 0.783, and it was found to be statistically significant (*p* < 0.01).

**TABLE 4:** Descriptive features and internal consistency reliability of Turkish HCAHPS.

	Number of items	Mean	SD	Observed range	Cronbach's alpha	McDonald's omega *ω*	Mean inter‐item correlation	Corrected item‐total correlation
Composites								
Communication with nurses	3	3.70	0.61	1–4	0.95	0.95	0.862	0.720–0.783
Communication with doctors	3	3.82	0.48	1–4	0.92	0.92	0.808	0.584–0.626
Responsiveness of hospital staff	2	3.42	0.60	1–4	0.74	0.74	0.590	0.531–0.652
Hospital environment	2	3.67	0.59	1–4	0.71	0.71	0.561	0.533–0.595
Communication about medicines	2	3.69	0.69	1–4	0.84	0.87	0.770	0.484–0.517
Discharge information	2	1.77	0.38	1–2	0.84	0.84	0.737	0.490–0.515
Care transition	3	3.45	0.70	1–4	0.83	0.84	0.628	0.550–0.721
Global ratings								
Overall rating of hospital	1	8.53	2.35	0–10	—	—	—	—
Willingness to recommend hospital	1	3.60	0.79	1–4	—	—	—	—

*Note:* Mean inter‐item correlation is reported for each multi‐item composite; for two‐item composites, this value equals the single inter‐item correlation. Corrected item‐total correlations are reported as the minimum–maximum range within each composite.

As part of the additional item‐level analyses, item discrimination was examined to determine each item's ability to distinguish between high‐ and low‐scoring respondents. Specifically, the mean scores of the upper 27% and lower 27% of respondents were compared using Student's *t*‐test for each item. Results showed a statistically significant difference between the mean scores of the upper group (*n* = 129) and the lower group (*n* = 129) for all items (*p* < 0.01), supporting adequate item discrimination across the scale.

## 4 Discussion

The present study aimed to adapt the HCAHPS survey to the Turkish healthcare context. The findings support the reliability and validation of the Turkish version of HCAHPS, indicating that it effectively assesses patient experiences across hospital settings. To further explore these insights, we discussed these findings about other international adaptation studies; we also highlighted the limited number of such studies and the need for more studies on cross‐cultural adaptations in different contexts worldwide.

### 4.1 Patient Experience Composites

The highest composite score in the current study was for communication with doctors, closely followed by communication with nurses. Similar patterns observed in Japan and the Philippines (Aoki et al. 2020; Judan‐Ruiz et al. 2020) suggest a shared strength in provider‐patient communication across various hospital settings internationally. This pattern is also seen for discharge information and the responsiveness of hospital staff, ranked as the lowest‐scoring areas in the Turkish context, and also in Japan and the Philippines (Aoki et al. 2020; Judan‐Ruiz et al. 2020). This highlights shared areas of concern across all three countries and indicates significant opportunities for improvement in patient care practices across these healthcare systems.

### 4.2 Validity

The content validity assessment, conducted with nine experts, confirmed that the survey items retained their conceptual relevance after translation. The I‐CVI and S‐CVI values exceeded the recommended thresholds, suggesting that the adapted version accurately captures the intended constructs. Similar results have been observed in other studies, where content validity indexes confirmed the relevance and appropriateness of the survey items. For example, the Filipino version of HCAHPS achieved a CVI of 1.00, indicating excellent conceptual and content equivalence (Judan‐Ruiz et al. 2020), and the Malay version reported a CVI of 0.87, with individual item indices ranging from 0.8 to 1.0 (Zun et al. 2019).

Further validation of the Turkish version was supported by CFA, which demonstrated that the factor structure remained stable after translation. Model fit indices (*χ* ^2^/df = 2.06, RMSEA = 0.05) indicated a good fit. This structural validity is consistent with results from other international adaptations of HCAHPS. For instance, the Japanese version showed excellent model fit indices (CFI = 0.987, RMSEA = 0.031) and factor loadings ranging from 0.41 to 0.91, indicating a robust structural framework (Aoki et al. 2020). The Malay version's exploratory factor analysis (EFA) reported factor loadings between 0.652 and 0.961, suggesting strong structural validity across domains (Zun et al. 2019). Additionally, the criterion‐related validity of the Turkish HCAHPS was confirmed through a significant positive correlation (*r* = 0.813, *p* < 0.01) between the composite scores and the overall hospital rating (Q18). Item discrimination also demonstrated that the survey items effectively differentiated between high and low scorers, further validating the instrument's ability to distinguish varying levels of patient experience.

### 4.3 Reliability

Regarding reliability, the Turkish HCAHPS exhibited satisfactory internal consistency, with Cronbach's alpha values ranging from 0.71 to 0.95, demonstrating that the survey items are strongly interrelated. These findings align with the majority of those of other HCAHPS versions. In the Japanese version, however, the internal consistency across domains showed a mixed profile. The domains related to communication with nurses and doctors showed acceptable reliability—with alpha values of 0.85 and 0.89, respectively—but considerably lower values, 0.46 and 0.49, were found for assessing responsiveness of hospital staff and hospital environment, respectively, indicating suboptimal internal consistency. Similarly, only moderate to low reliability was found for the domains for communication about medicines and discharge information, with alpha values of 0.71 and 0.48, respectively. In contrast, in some other contexts, higher values were recorded. The Malay version reported domain values ranging from 0.68 to 0.92 and an overall survey value of 0.84 (Zun et al. 2019), the Filipino version demonstrated an alpha of 0.85 (Judan‐Ruiz et al. 2020), and the Arabic HCAHPS showed an overall alpha of 0.90 (Alanazi et al. 2017). Additionally, the internal consistency of the Turkish HCAHPS was further supported by item‐total correlation values between 0.484 and 0.783, confirming that all survey items correlate strongly with the overall score. Overall, these findings confirm the survey's strong reliability across a wide range of cultural and linguistic settings.

### 4.4 Strengths and Limitations

One of the strengths of this study is the rigorous translation and cultural adaptation process, which adhered to established guidelines (AHRQ 2016). The use of multiple independent translators, expert review, and reconciliation ensured linguistic and conceptual accuracy. Additionally, the generalizability of the findings is enhanced by the inclusion of a diverse sample from three private hospitals with differing characteristics in different locations. However, this study has some limitations. First, the sample consisted exclusively of patients from private hospitals, which may limit the applicability of the findings to public healthcare settings. Future research should consider validating the instrument in public hospitals to ensure broader applicability.

## 5 Conclusion

This study describes how the HCAHPS survey was successfully adapted for use in Turkey and revealed its validation features. The Turkish version demonstrated strong content validity, meeting the recommended thresholds. It also maintained its original structure following the content validity assessment. CFA indicated good fit indices and factor loadings, confirming the survey's robust construct validity. Additionally, the Turkish HCAHPS's criterion‐related validity was supported by its significant positive correlation with the overall hospital rating. Reliability was demonstrated through statistically significant Cronbach's alpha, McDonald's omega, and item‐total correlation values. These findings support the HCAHPS survey's global applicability and confirm its status as a valuable tool for evaluating patient experiences. Hospital managers and healthcare providers implementing the Turkish version can effectively assess patient‐centered care and make improvements accordingly. This study contributes to the growing body of evidence, confirming that the HCAHPS survey can be successfully adapted across different cultural and linguistic contexts while maintaining its integrity as a reliable and valid instrument for measuring patient experiences.

### 5.1 Relevance for Clinical Practice

The validated Turkish HCAHPS will be essential for measuring patient experiences and enhancing hospital services. Insights gathered from patient‐reported experiences can guide hospital policies and quality improvement initiatives, enabling healthcare administrators and policymakers to strengthen communication, responsiveness, and overall patient‐centered care. This approach aligns with global trends, as exemplified in the United States, where public reporting of HCAHPS has significantly improved patient care, particularly in hospitals that initially received lower scores (Elliott et al. 2015). Similar advancements can be expected in Turkey and other countries when HCAHPS data is utilized to enable healthcare providers to identify areas needing improvement.

The HCAHPS survey can pinpoint specific opportunities for enhancing patient experiences across various healthcare settings, including hospitals and long‐term care facilities. One crucial area for improvement that has been identified, especially in hospitals with lower patient experience scores, is the work environment of healthcare staff. Improvements in work conditions can lead to better communication among healthcare providers, more effective pain management, and reduced response times, directly affecting patient experience. This phenomenon has also been observed in countries such as Chile, where better work environments were found to lead to improved patient experience (Simonetti et al. 2023). The HCAHPS survey offers a valuable framework for healthcare providers worldwide to enhance working environments and thus patient experiences.

Future research could consider the example of the Turkish HCAHPS and explore the scale's further application across regions and countries worldwide, extending its adaptation to diverse cultural and healthcare systems. Longitudinal studies could assess the instrument's sensitivity in detecting changes in patient experience over time. Given the increasing global emphasis on patient‐centered care, the availability of a validated HCAHPS in other languages in addition to Turkish can be a significant resource for advancing healthcare research and practice. This will enable a more comprehensive approach to improving patient care in various settings, within Turkey and beyond.

## Author Contributions

**Mehmet Gülşen:** conceptualization, methodology, supervision, formal analysis, writing – original draft, writing – review and editing, visualization, validation, funding acquisition, project administration. **Hasan Fehmi Dirik:** conceptualization, methodology, validation, supervision, writing – review and editing, writing – original draft. **Kubilay Kaymaz:** conceptualization, funding acquisition, investigation, writing – review and editing. **Asuman Kuşçu:** data curation, investigation, writing – original draft, conceptualization, funding acquisition. **Melis Yazici:** conceptualization, funding acquisition, writing – review and editing, investigation. **Işıl Yerlikaya:** conceptualization, investigation, funding acquisition, writing – review and editing. **İlkay Baylam:** conceptualization, investigation, funding acquisition, data curation, writing – review and editing.

## Funding

This study was supported by Balıkesir University—Scientific Research Projects Unit under Project No 2023/087.

## Ethics Statement

Permission to translate the HCAHPS into Turkish was first obtained from the AHRQ CAHPS Program and the U.S. CMS. Subsequently, ethical approval for this study was granted by the non‐interventional research ethics committee of Balıkesir University (Decision Date: March 7, 2023, Decision Number: 2023/29).

## Consent

Informed consent has been obtained from all patients prior to the start of the study. Written permissions to administer the survey were also obtained from the hospitals.

## Conflicts of Interest

Hasan Fehmi Dirik is an Associate Editor for Nursing & Health Sciences and the author of this article. The manuscript was managed by editors unaffiliated with the author or institution and monitored carefully to ensure there is no peer review bias. The other authors declare no conflicts of interest.

## Data Availability

The data that support the findings of this study are available from the corresponding author upon reasonable request.
